# On the Relationship Between Oceanic Plate Speed, Tectonic Stress, and Seismic Anisotropy

**DOI:** 10.1029/2022GL097795

**Published:** 2022-08-10

**Authors:** E. Kendall, M. Faccenda, A. M. G. Ferreira, S.‐J. Chang

**Affiliations:** ^1^ Department of Earth Sciences University College London London UK; ^2^ GFZ German Research Centre for Geosciences Potsdam Germany; ^3^ Dipartimento di Geoscienze Università di Padova Padua Italy; ^4^ CERIS Instituto Superior Técnico Universidade de Lisboa Lisbon Portugal; ^5^ Department of Geophysics Kangwon National University Chuncheon South Korea

**Keywords:** anisotropy, oceanic lithosphere, upper mantle, geodynamic modeling, deformation

## Abstract

Seismic radial anisotropy (the squared ratio between the speeds of horizontally and vertically polarized shear waves, $\xi =\frac{{V_{SH}}^{2}}{{V_{SV}}^{2}}$) is a powerful tool to probe the direction of mantle flow and accumulated strain. While previous studies have confirmed the dependence of azimuthal anisotropy on plate speed, the first order control on radial anisotropy is unclear. In this study, we develop 2D ridge flow models combined with mantle fabric calculations to report that faster plates generate higher tectonics stresses and strain rates which lower the dislocation creep viscosity and lead to deeper anisotropy than beneath slower plates. We apply the SGLOBE‐rani tomographic filter, resulting in a flat depth‐age trend and stronger anisotropy beneath faster plates, which correlates well with 3D global anisotropic mantle models. Our predictions and observations suggest that as plate speed increases from 2 to 8 cm/yr, radial anisotropy increases by ∼0.01–0.025 in the upper 100–200 km of the mantle between 10 and 60 Ma.

## Key Points

- Faster oceanic plates produce higher tectonic stresses, lower dislocation creep viscosities, and therefore deeper radial anisotropy
- As plate speed increases from 2 to 8 cm/yr, recovered *ξ* increases by ∼0.01–0.025 in the upper 100–200 km of the mantle between 10 and 60 Ma
- The observed flat depth‐age trend in radial anisotropy tomography models is likely due to strong regularization

## 1 Introduction

The asthenosphere is a ductile region which experiences large deformation and is a key control on present day plate motions. It primarily deforms in both the dislocation and diffusion creep regime (Karato, 1993). Diffusion creep is characterized by the diffusion of vacancies and is thought to not produce a lattice‐preferred orientation (LPO) except under certain conditions of temperature and in the presence of melt (Miyazaki et al., 2013). On the other hand, dislocation creep occurs by the slipping along crystallographic glide planes and results in LPO. Large scale seismic anisotropy in the upper mantle has long been attributed to this alignment of mineral grains by large‐strain deformation such as by plate motions (Debayle et al., 2005; Karato, 2008; Nicolas & Christensen, 1987; Vinnik et al., 1992; Zhang & Karato, 1995). In most cases, to first order at shallow mantle depths, *V*_SH_ > *V*_SV_ or *ξ* > 1 (*V*_SV_ > *V*_SH_ or *ξ* < 1) indicates horizontal (vertical) flow in the upper mantle.

Seismic anisotropy is largely determined by two parameters: transition stress (the stress at which both dislocation and diffusion creep accommodate an equal amount of strain) and tectonic stress (Podolefsky et al., 2004). For example, dislocation (diffusion) creep is dominant when tectonic stress is above (below) the transition stress (Hirth & Kohlstedt, 2003; Karato, 1993). In the asthenosphere, the relative motion of the overlying plate controls (to first order) strain rate and viscosity, and therefore tectonic stress and the creep regime. Another mechanism that can lead to seismically observable anisotropy is shape‐preferred orientation (Wang et al., 2013) from melt pockets (Kendall, 1994; Mainprice, 1997), while Faccenda et al. (2019) demonstrated that compositional layering produces negligible seismic anisotropy in the upper mantle.

The first correlation between seismic anisotropy and plate speed was highlighted by Wolfe and Solomon (1998), in which they found that the magnitude of azimuthal anisotropy beneath the faster‐moving Pacific Plate is twice that of the slower Nazca plate. Debayle and Ricard (2013) later reported a strong correlation between azimuthal anisotropy and absolute plate motion in the asthenosphere. In terms of radial anisotropy, Auer et al. (2015) conducted a series of forward calculations and also visualized the LPO‐based *ξ* predictions of Becker et al. (2008) for A‐type fabric to report that the Pacific asthenosphere required stronger and deeper radial anisotropy compared to the Atlantic. And while there have been many regional studies focused on the Pacific (Kendall et al., 2021) and Atlantic (Silveira & Stutzmann, 2002) upper mantle structure, the relationship between radial anisotropy and oceanic plate speed has not yet been fully investigated.

The polycrystal deformation model, D‐Rex (Kaminski et al., 2004) can compute deformation‐induced LPO, and has been extensively used to predict seismic anisotropy in the oceanic upper mantle (Becker et al., 2008; Gallego et al., 2013; Hedjazian et al., 2017). In this study, for the first time, we present comparisons of asthenospheric radial anisotropy beneath different oceans from current 3D global anisotropic mantle models and 2D ridge flow models with fabric calculations. We also expand on previous studies, such as that of Hedjazian et al. (2017) by applying a recent tomographic filter to the synthetic seismic structure.

Specifically, we address the following questions: (a) Is there a correlation between magnitude of radial anisotropy and plate speed? (b) If so, can an increase in plate speed relative to the underlying asthenosphere explain this observation? By doing so we can more accurately interpret seismic images beneath the relatively simple oceanic settings and better understand how tectonic stresses control asthenospheric deformation which in turn affects plate dynamics. While isotropic structure shows a depth‐age trend, radial anisotropy is imaged to be age‐independent (Beghein et al., 2019) with average isotropy persisting from ∼200 km depth. It has been proposed that the presence of melts and/or fluids at lithosphere‐asthenosphere boundary depths (Beghein et al., 2014; Burgos et al., 2014) may be responsible for this observation. Therefore, the final question we address is: (c) What controls the flattening of the depth‐age trend in radial anisotropy?

## 2 Seismic Observations

Fundamental‐mode surface‐wave dispersion data have strong sensitivity to the upper mantle (Chang et al., 2014). Therefore, we choose to analyze the three following 3D global anisotropic mantle models which were built using millions of fundamental‐mode surface wave measurements: (a) S362WMANI (Kustowski et al., 2008), (b) SGLOBE‐rani (Chang et al., 2015) and (c) SAVANI (Auer et al., 2014). These models have been built using similar datasets, the same theoretical framework (ray theory) but different parametrizations and have accounted for the crust in different ways. S362WMANI was one of the first studies to implement an alternative spherically symmetric reference model than PREM and to simultaneously invert for velocity and anisotropy. In order to reduce the effects of the crust on the mapping of radial anisotropy in the mantle (Ferreira et al., 2010), Chang et al. (2015) jointly inverted for 3D isotropic and radially anisotropic structure, as well as for crustal thickness variations with respect to CRUST2.0, obtaining the model SGLOBE‐rani. S362WMANI uses spherical splines and B‐splines and SGLOBE‐rani uses spherical harmonic basis functions and depth spline functions to parametrize variations in the horizontal and radial direction, respectively. The final model we include in this study is SAVANI, which uses variable blocks for horizontal variations, adapted to local raypath density and layers to parametrize variations in the radial direction. For further details on the construction of these models please see the respective papers.

**Figure 1:**
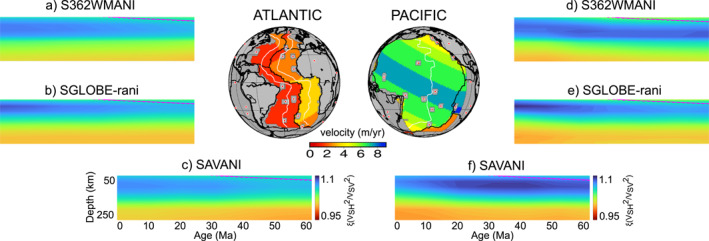
Radial anisotropy in (a) S362WMANI (Kustowski et al., 2008), (b) SGLOBE‐rani (Chang et al., 2015), and (c) SAVANI (Auer et al., 2014) as a function of ocean‐sea floor age beneath the Atlantic (left) for profiles within the white lines (up to 60 Ma) in the map of observed plate motion (NUVEL‐1A in a no‐net‐rotation frame from DeMets et al. (2010)). Profiles are removed within 3° each of each plume (red circles) in the Sleep (1990) hotspot list. Cross‐sections beneath the Pacific can be found on the right for the same tomographic models (d–f), respectively. The 1,000°C isotherm from the half‐space cooling model is shown by a dashed/solid magenta line.

Figures 1a–1c and Figure S1 in Supporting Information S1 of the supplementary materials shows radial anisotropy beneath the Atlantic and the Indian ocean, respectively, as imaged by S362WMANI, SGLOBE‐rani, and SAVANI. Note that we remove profiles 3° each side of each plume in the Sleep (1990) hotspot list to reduce any plume‐related anisotropy. The Atlantic and Indian plates are moving on average at 2 and 5 cm/yr, respectively. The Atlantic and Indian asthenosphere is characterized by *ξ* > 1, indicating, to first order, horizontal flow. On the other hand, the Pacific plate is one of the fastest in the world with an average speed of ∼8 cm/yr. The strength of radial anisotropy increases with plate speed (Figure 1). Radial anisotropy reaches a maximum of *ξ* ∼ 1.06–1.07 at ∼75–100 km depth beneath 30 Ma lithosphere of a slow plate (Figures 1a–1c) as opposed to *ξ* ∼ 1.07–1.09 at ∼75–125 km depth beneath fast oceanic plates (Figures 1d–1f).

To verify the correlation between the strength of radial anisotropy and plate speed we quantify the seismic resolution by computing Backus–Gilbert averaging kernels for SGLOBE‐rani for an oceanic region in the Atlantic, Indian ,and Pacific at a depth of 74 and 129 km (Figure S2 in Supporting Information S1). These kernels describe how the velocity or anisotropy perturbation at a given point is a spatial average of the real structure. In practice, Backus–Gilbert kernels deviate from delta functions and have a finite spatial extent due to heterogeneous data coverage, model regularization and the finiteness of model parametrization. Figure S2 in Supporting Information S1 shows cross‐sections through these kernels. Overall, the anisotropic structure (dashed lines) are similarly well resolved beneath each oceanic plate and thus it is fair to compare them.

## 3 Geodynamic Mantle Modeling and Fabric Calculations

### 3.1 2D Ridge Flow Models

To simulate surface‐driven mantle flow beneath an oceanic plate, we solve the compressible Stokes, continuity and heat diffusion equations in polar coordinates using the finite difference code I2VIS (Gerya & Yuen, 2003). Figure S3 in Supporting Information S1 shows a schematic of the 2D polar domain: 40° in longitude by 700 km in depth. It contains a ridge‐axis on the left‐hand side and a subduction zone aided by a weak zone with a fixed dip angle of 45° on the right. Moreover, we have a mesh resolution of 1 × 1 km at the ridge and increasing to 4 and 2 km along the longitudinal and vertical directions, respectively.

**Figure 2:**
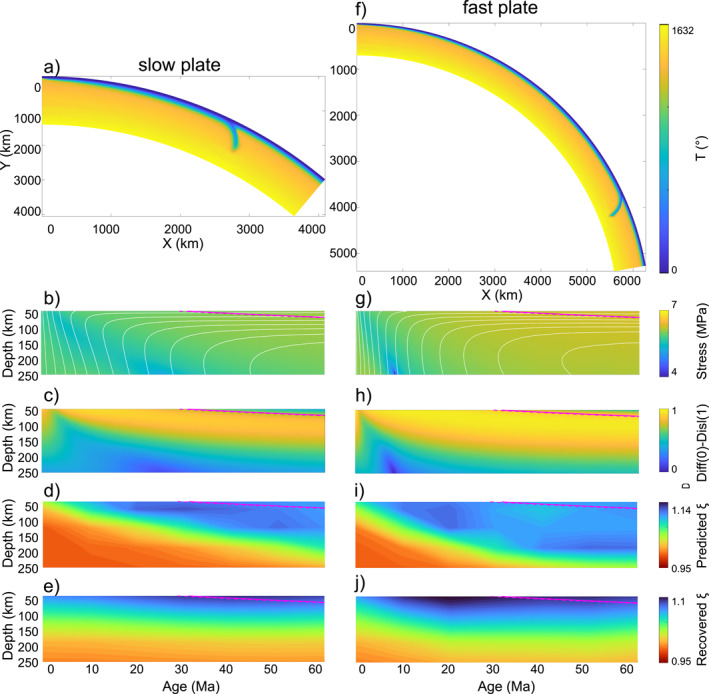
(a) Temperature field showing the half‐space cooling solution overlying an adiabat gradient increasing from 1,540 K at 0.5 K/km to 1,890 K at 700 km, (b) stress and streamlines (white lines), (c) deformation mechanism (the fraction of deformation accommodated by dislocation creep is computed as *η*_eff_/*η*_disl_ and it varies from 0 (*η*_disl_ ≫ *η*_eff_; i.e., no dislocation creep) to 1 (*η*_disl_ = *η*_eff_; Sturgeon et al. (2019)), (d) predicted radial anisotropy (which is not smoothed before the application of the tomographic filter) and (e) tomographically filtered predictions of radial anisotropy for the slow plate model (2 cm/yr). (f–j) Same as (a–e) but for the fast plate (8 cm/yr) with the rheological parameters found in Table S1 in Supporting Information S1. The 1,000°C isotherm from the half‐space cooling model is shown by the dashed/solid magenta line.

Initial conditions include a fixed cold temperature (273 K) for the 25 km thick sticky‐air layer imposed at the surface to simulate the free‐surface, a conductive oceanic plate following the half‐space cooling model and an adiabatic gradient of 0.5 Km^−1^ in the underlying hot mantle (Figures 2a and 2b). All sides are prescribed free slip conditions except the bottom which is permeable. In order to investigate the effect of plate velocity on radial anisotropy in the upper mantle we apply a constant velocity of 2 and 8 cm/yr to represent slow and fast plates, respectively. For the latter plate speed we therefore extend the domain from 40° longitude to 80° to account for the faster motion.

Deformation is accommodated by a visco‐plastic rheological model as defined in Section S1.1 in Supporting Information S1. The reference rheological parameters properties used to calculate viscosity (Figures S6b and S6d in Supporting Information S1) can be seen in Table S1 and S2 in Supporting Information S1. These parameters are widely used and have been extensively tested in previous studies (Yang & Faccenda, 2020).

### 3.2 Mantle Fabrics Calculations

Following the same procedure as for example, Ferreira et al. (2019) and Sturgeon et al. (2019), we model the development of LPO using a modified version of the kinematic model D‐Rex (Kaminski et al., 2004) to simulate plastic deformation and dynamic recrystallization. We consider harzburgitic aggregates with 70% olivine and 30% enstatite in the upper mantle while pyrolitic aggregates in the transition zone depths are assumed to be isotropic. Aggregates are initially spaced 10 by 10 km in the tangential and radial directions, respectively. We extensively tested a wide range of the deformation parameters listed in Table S3 in Supporting Information S1 (Figures S4 and S5 in Supporting Information S1 which are discussed in Section S1.2 in Supporting Information S1). Following these tests, we choose the normalized reference shear stresses indicated in the top row of Table S3 in Supporting Information S1 with the grain boundary sliding parameter, *χ* = 0.9 and a grain boundary mobility, M* of 1, which result in fabrics analogous to the AG‐type, although displaying some weak azimuthal anisotropy. These parameters have been shown to be more consistent with both radial and azimuthal anisotropy observations in intra‐oceanic settings and trench‐parallel SKS anisotropy at forearcs than the more conventional A‐type fabric (Rappisi & Faccenda, 2019; Figures S4 and S5 in Supporting Information S1 and also some additional calculations presented in the Discussion). Love parameters are calculated directly from the Voigt averaged tensor (Montagner & Nataf, 1986) as discussed in the supplementary materials.

### 3.3 Predictions of Oceanic Upper Mantle Anisotropy

Figures 2b–2d shows the stress, dominant deformation mechanism and predicted radial anisotropy beneath the slow plate with rheological parameters from Table S1 in Supporting Information S1. Dislocation creep is the predominant deformation mechanism in the top several tens of kilometres beneath the plate. Beneath the ridge pure shear deformation is diffused, while away from the ridge, simple shear deformation dominates (Figures S6c and S6f in Supporting Information S1) and mantle flow is sub‐horizontal leading to positive radial anisotropy, *ξ* > 1. On the other hand, faster plate motion (Figures 2g–2i) induces higher tectonic stresses and strain rates, resulting in a lower dislocation creep viscosity (Figures S6b and S6e in Supporting Information S1) and deeper radial anisotropy than beneath slow plates (Figure S7 in Supporting Information S1).

### 3.4 Tomographic Recovery

For a fair comparison between the mantle fabrics calculations and the tomography models we project the synthetic seismic anisotropy as seismic images using the SGLOBE‐rani tomographic filter. We thereby take into account the finite seismic resolution due to incomplete data coverage, the model parametrization and regularization typically used in global tomography. The synthetic seismic structure is projected into the parametrization of SGLOBE‐rani and convolved with the resolution filter of SGLOBE‐rani (Ritsema et al., 2007; Styles et al., 2011; see Section S1.3 in Supporting Information S1 for further details). Application of the tomographic filter to the geodynamic model corresponds to performing a synthetic inversion with the same ray coverage, forward modeling and inversion approach as used in SGLOBE‐rani.

The depth‐age dependency in the predictions of radial anisotropy is not recovered when applying the tomographic filter Figure 2e, showing similarities to the flat signature in tomography models (Figures 1a–1c). The poorer resolution of radial anisotropy with increasing depth (Figure S8 in Supporting Information S1) is consistent with the broader Backus‐Gilbert kernels at greater depths (e.g., Figure S2 in Supporting Information S1).

Beneath the fast plate (Figure 2j), stronger radial anisotropy persists than beneath the slow plate. Recovered radial anisotropy beneath the slow plate reaches a maximum of *ξ* ∼ 1.07–1.09 at ∼50–80 km depth beneath 30 Ma, correlating well with tomography models beneath the Atlantic (Figures 1a–1c). Recovered radial anisotropy beneath the fast plate reaches a maximum of *ξ* ∼ 1.08–1.1 at ∼50–100 km depth beneath 30 Ma, correlating well with tomography models beneath the Pacific (Figures 1a–1c).

**Figure 3:**
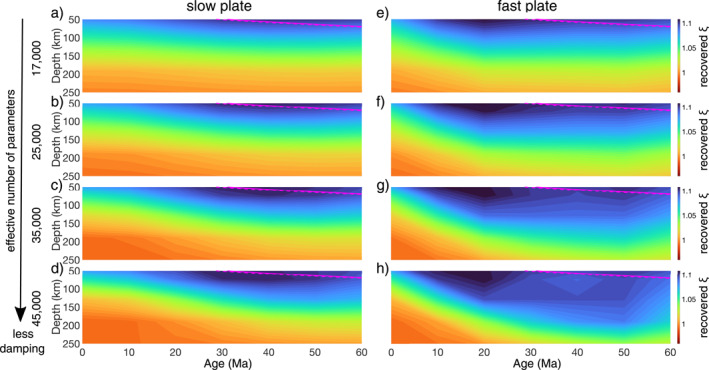
Recovered *ξ* as a function of ocean‐sea floor age beneath the slow plate for the reference model with the rheological parameters found in Table S1 in Supporting Information S1 with effective number of model parameters (a) 17,000 (most damping), (b) 25,000, (c) 35,000, and (d) 45,000 (least damping). (e–h) Same as (a–d) but for the fast plate. The 1,000°C isotherm from the half‐space cooling model is shown by the dashed/solid magenta line.

Limited data coverage and bandwidth and/or strong regularization are the most likely causes of the flattened depth‐age trend. Figure 3 shows that a depth‐age trend can be recovered with weak regularization, indicating that the flattening may be at least partly due to the strong regularization typically used in global tomography inversions to stabilize them.

**Figure 4:**
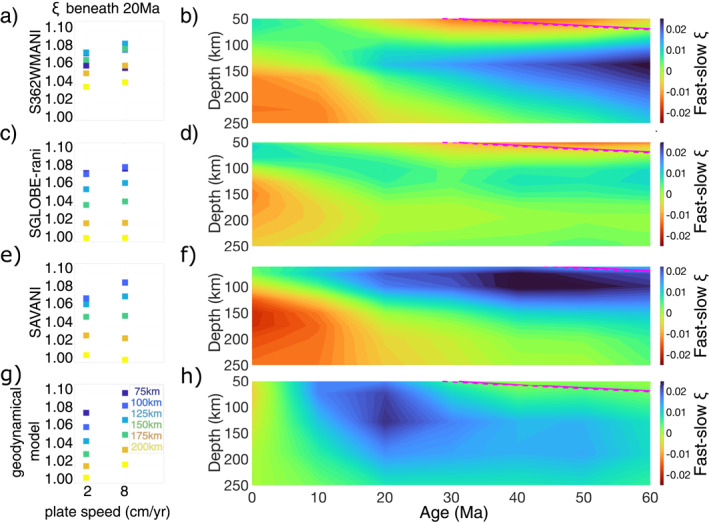
(a) The dependence of radial anisotropy (*ξ*) on plate speed and (b) the difference in the observed anisotropy beneath fast (Pacific) and slow (Atlantic) plates for S362WMANI. The same for format can be seen for: SGLOBE‐rani (c, d), SAVANI (e, f) and the tomographically filtered geodynamical model presented in this study (g, h).

Predictions of the dependence of radial anisotropy on plate speed correlate well with observations (Figure 4). While there are differences in the magnitude and depth extent of radial anisotropy between the different tomography models, as plate speed increases from 2 to 8 cm/yr, observed and predicted radial anisotropy increase by ∼0.01–0.025 in the upper 100–200 km of the mantle between 10 and 60 Ma (Figures 4b, 4d, 4f and 4h). Observations of radial anisotropy directly beneath the ridge (Figures 4b, 4d and 4f) show a reduction in radial anisotropy and therefore to first order more sub‐vertical deformation for faster plates. The predictions of radial anisotropy (Figure 4h) do not reproduce this pattern potentially because of the kinematic boundary conditions that prevent the formation of strong mantle fabrics in the isotropic mantle aggregates upwelling from the transition zone. Moreover, we also note that the location at which the maximum difference between the radial anisotropy beneath the fast and slow plate occurs is different in the geodynamical and seismological models. This may be related to (a) the use of different data sets, parameterizations and regularization utilized for the different tomographic models, (b) uncertainties in mantle rheology, (c) plume‐induced effects which have not been entirely removed, and/or (d) melt‐pockets (Kawakatsu et al., 2009).

## 4 Discussion

Our results are in agreement with Podolefsky et al. (2004), who found that slower plate motion leads to a thinner layer of dislocation creep with positive radial anisotropy. Figures S9 and S10 in Supporting Information S1 show that a depth‐age trend in isotropic shear wave velocity can be recovered, unlike for radial anisotropy. While we do not rule out that the presence of melts and/or fluids at lithosphere–asthenosphere boundary depths may be partly responsible for a flat depth‐age trend in radial anisotropy, we have shown that strong tomographic regularization is a key factor. This is also in agreement with Beghein et al. (2019). To better understand the discrepancies between other global tomography models and the recovered structures from the geodynamic models, one would need to apply the tomographic filter of the other models, which are not available. Hedjazian et al. (2017) predict a flat depth‐age dependency of radial anisotropy as opposed to this study. The flow field in Hedjazian et al. (2017) is influenced by the velocity boundary condition applied at the top and right boundaries, which results in low deformation rates in the asthenosphere away from the ridge. Moreover, we compute viscosities two orders of magnitude lower than in Hedjazian et al. (2017) which focuses deformation within the shallow asthenosphere (Figures S6b and S6e in Supporting Information S1).

S362WMANI, SGLOBE‐rani, and SAVANI show a decrease in radial anisotropy with age beneath the Atlantic plate, unlike beneath the Pacific as imaged by S362MWANI, SAVANI or our predictions. Small‐scale convection (SSC) could rotate fast axes away from the horizontal direction, decreasing the observed positive radial anisotropy. However, in this study the domain is two‐dimensional and the lithosphere is likely too young, ∼60 Ma, to generate significant SSC (Coltice et al., 2018). Moreover, the higher effective viscosity in the asthenosphere beneath the Atlantic than beneath the Pacific would lead to an increase in the onset time of SSC (Ballmer et al., 2010). Accordingly, we propose that plume–lithosphere interactions, which may not have been entirely removed in our compilation, may be the most important mechanism responsible for the destruction of *ξ* > 1 textures beneath the Atlantic plate. Specifically, where plumes rise and spread beneath oceanic lithosphere the flow lines and therefore fast axes of the FSE are less coherent in the horizontal direction (d’Acremont et al., 2003). In addition, in the Pacific plate motion relative to the asthenosphere is likely driven by slab pull. This condition is not met in the Atlantic where the oceanic plate could be more coupled with the asthenosphere, leading to lower strain rates and anisotropy. Moreover, future studies should investigate the effect of different ridge systems such as in the south Pacific, and of asthenospheric mantle flow at an angle from the plate motion, via 3D modeling.

Predictions of radial anisotropy in this study are stronger in the lithosphere than in the tomographic models. However, global tomography models such as those discussed in this study have limited resolution at lithospheric depths (Ferreira et al., 2010) beneath the oceans and regional studies (Russell et al., 2018) indicate that *ξ* ≥ 1.05 in the uppermost (0–30 km) lithosphere as opposed to isotropy or *ξ* < 1 in global tomography models. Using dynamic models with no imposed plate motion will likely lead to lower tectonic stresses and less positive radial anisotropy, for example, in the lithosphere.

While the predictions of radial anisotropy presented in this study fit seismic observations well, there may be other D‐Rex parameter choices that lead to similar LPO patterns. Moreover, it should be noted that seismic anisotropy does not depend linearly on finite strain accommodated by dislocation creep (Tommasi et al., 2000) and, although we have used the parameters from Karato and Wu (1993), which have been widely tested, diffusion and dislocation creep parameters are not tightly constrained (Korenaga & Karato, 2008). Similar uncertainties exist for the activities of slip systems in mantle minerals. Therefore, for completeness we have also explored the dependence of radial anisotropy on a lower *V*_diffusion_ of 2 cm^3^/mol and A‐type olivine fabric (Figure S11 in Supporting Information S1). However, the former generates radial anisotropy that is too weak beneath a slow plate and the latter generates anisotropy that is too strong in comparison to observations.

## 5 Conclusions

We developed simple 2D ridge flow models combined with fabric calculations showing that faster plates generate higher tectonics stresses and strain rates which in turn lower the dislocation creep viscosity and lead to deeper anisotropy than beneath slower plates. We applied the tomographic filter of a recent tomographic model (SGLOBE‐rani) to our predictions and found that strong regularization is likely a key control of the flattening of a depth‐age trend in radial anisotropy. Our tomographically filtered predictions correlate well with observations from 3D global anisotropic mantle models, such that as plate speed increases from 2 to 8 cm/yr, radial anisotropy increases by ∼0.01–0.025 in the upper 100–200 km of the mantle between 10 and 60 Ma.

## Supporting information

Supporting Information S1

## Data Availability

The ridge flow models were built using I2VIS (Gerya & Yuen, 2003), which is not freely available but can be provided by Prof. T. Gerya via email request (taras.gerya@erdw.ethz.ch). Fabric calculations were carried out using a modified version of D‐Rex, which is included in the open source package ECOMAN (https://newtonproject.geoscienze.unipd.it/ecoman/).
