# Supplementary material for: On the Relationship Between Oceanic Plate Speed, Tectonic Stress, and Seismic Anisotropy
Source: Geophys Res Lett. 2022 Aug 10;49(15):e2022GL097795. doi: 10.1029/2022GL097795 (PMC9539886; doi:10.1029/2022GL097795)
Supplement: Supplementary file 1 — Supporting Information S1 [file GRL-49-e2022GL097795-s001.pdf]

Supplementary Information for "On the relationship between oceanic plate speed, tectonic stress and seismic anisotropy"

E. Kendall[1,2], M. Faccenda[3], A.M.G. Ferreira[1,4], S.-J. Chang [5]

[1] Department of Earth Sciences, University College London, London, UK

[2] GFZ German Research Centre for Geosciences, Potsdam, Germany

[3] Dipartimento di Geoscienze, Università di Padova, Padua, Italy

[4] CERIS, Instituto Superior Técnico, Universidade de Lisboa, Lisbon, Portugal

[5] Department of Geophysics, Kangwon National University, Chuncheon, South Korea

Contents of this file

1. Text
2. Figures S1 to S12

S1 Geodynamic mantle modeling and fabric calculations

S1.1 2D ridge flow models

I2VIS (Gerya and Yuen, 2003) has been modified to solve the equations for conservation of mass (eq.1), momentum (eq.2-3) and energy (eq. 4) in polar coordinates. In a Lagrangian reference frame and assuming incompressibility these equations take the form:

$$\frac{1}{r}\frac{\partial r v_r}{\partial r} + \frac{1}{r}\frac{\partial v_\phi}{\partial \phi} = 0 \quad (1)$$

$$\phi_{mom} : \frac{1}{r}\frac{\partial \tau_{\phi\phi}}{\partial \phi} + \frac{1}{r^2}\frac{\partial r^2 \tau_{\phi r}}{\partial r} - \frac{1}{r}\frac{\partial P}{\partial \phi} = 0 \quad (2)$$

$$r_{mom} : \frac{1}{r}\frac{\partial \tau_{r\phi}}{\partial \phi} - \frac{1}{r^2}\frac{\partial r^2 \tau_{\phi\phi}}{\partial r} - \frac{\partial P}{\partial r} = -\rho g_r \quad (3)$$

$$\rho C_p \frac{DT}{Dt} = -(\frac{1}{r}\frac{\partial r q_r}{\partial r} + \frac{1}{r^2}\frac{\partial q_\phi}{\partial \phi}) + H \quad (4)$$

where ϕ and r are the tangential and radial coordinates, respectively, $\overrightarrow{v}$ is the velocity vector, P is pressure, τ is the deviatoric stress tensor, ρ is density, g_r is the radial gravitational acceleration (9.81 m s^{-2}), C_p is the specific heat capacity, T is the temperature, D/Dt is the Lagrangian time derivative, $\overrightarrow{q}$ is the heat flux and H is a heat source term accounting for radiogenic, adiabatic and shear heating.

Viscosity at high-T is calculated from the general power law (e.g., Karato, 1993; Hirth and Kohlstedt, 2003):

$$\eta_{disl,diff} = \frac{1}{2}\dot{\varepsilon}_{II}^{\frac{1}{n}-1} A^{-\frac{1}{n}} \mu \left(\frac{d}{b}\right)^{\frac{m}{n}} \exp^{\frac{E+PV}{nRT}} \quad (5)$$

where $\dot{\varepsilon}_{II}$ is the second invariant of the strain rate tensor, n is the stress exponent, A is a prefactor, μ is the shear modulus, b is the length of the Burgers vector, d is the grain size, m is the grain size exponent, E is the activation energy, P is the total pressure, V is the activation volume, R is the gas constant and T is the temperature.

At high deviatoric stresses (greater than 0.1 GPa) and low-T conditions, creep is accommodated via the Peierls mechanism as given by Katayama and Karato (2008):

$$\eta_{peierls} = 0.5A\tau_{II}^{-1}exp\left\{\frac{E+PV}{RT}\left[1-\left(\frac{\tau_{II}}{\sigma_{Peierls}}\right)^{p}\right]^{q}\right\}., \tag{6}$$

where τ_{II} is the second invariant of the deviatoric stress tensor and all the other parameters are defined in Table S1. The effective, isotropic viscosity is the mean of the combined dislocation (power law), diffusion (newtonian) and Peierls creep viscosities, $(\eta_{ductile} = \eta_{\mathrm{disl}}^{-1}+\eta_{\mathrm{diff}}^{-1}+\eta_{\mathrm{peierls}}^{-1})^{-1}$.

A pseudo-plastic viscosity is also computed as:

$$\eta_{pl} = \frac{\tau_y}{2\dot{\varepsilon}_{II}}\ , \tag{7}$$

where $\dot{\varepsilon}_{II}$ is the second invariant of the strain rate and the plastic strength τ_y is determined with a plastic Drucker–Prager criterion:

$$\tau_y = C_{DP} + \mu P \tag{8}$$

where $C_{DP} = C\cos\phi = 1MPa$ is the cohesion, $\mu = \sin\phi$ is the friction coefficient and ϕ is the friction angle. To model strain-induced brittle weakening, the initial friction 0.6 coefficient is linearly decreased to 0.4 in the $0.5 \leq \varepsilon_p \leq 1.5$ range, where ε_p is the accumulated brittle/plastic strain. For the crust we use a constant $\mu = 0.05$ to ensure lubrication at the plate's contact. Finally, the effective viscosity is given by:

$$\eta_{eff} = min(\eta_{ductile}, \eta_{pl}) \tag{9}$$

The lower and upper cutoff of η_{eff} are set to 10^{18} and 10^{25} $Pa\ s$, respectively.

Pressure-temperature dependent density and enthalpy maps are generated using PERPLE_X for a pyrolitic mantle composition (Mishin et al., 2008) which allows to account also for phase transitions.

A curved geometry is more applicable to Earth than a box and is more appropriate in order to compare the geodynamical models with tomography models. However, in practice there is little difference in the results between the two geometries. Including a crust in these

models creates a more realistic oceanic plate rheology that lubricates the plates' contact during subduction. Without this weak layer the upper plate would be dragged downward yielding a double-sided subduction zone instead of a more realistic one-sided subduction zone (e.g., Gerya et al., 2008). We impose a subduction zone within the modelling domain to replicate the Pacific setting. Moreover, the initiation of subduction in geodynamical models is an ongoing area of research and traditionally a weak layer is imposed to mimic a weak interface at a plate boundary and initiate subduction.

S1.2 Mantle fabrics calculations

The modified version of D-Rex employed in this study uses time-dependent velocity fields and forward advection of crystal aggregates in order to account for the non-steady-state flow (Faccenda and Capitanio, 2013). However, for each model we have used a single velocity field representative of a mature and steady-state stage of the oceanic plate evolution from ridge to the subduction zone. The external velocity gradient tensor can be computed from the velocity field and then decomposed into a symmetric strain rate tensor (internal deformation rate) and an anti-symmetric vorticity tensor (rotation rate of the macroscopic flow field). Once all deformation is taken up internally, the magnitude and orientation of the transverse isotropy axis is extrapolated from the full elastic tensor (scaled by pressure and temperature) using the projection method of Browaeys and Chevrot (2004) providing a rapid estimate of the elastic anisotropy.

Love and azimuthal anisotropy parameters, are calculated directly from the Voigt averaged tensor (Montagner and Nataf, 1986):

$$L = \frac{1}{2}(C_{44} + C_{55}) \tag{10}$$

$$N = \frac{1}{8}(C_{11} + C_{22}) - \frac{1}{4}C_{12} + \frac{1}{2}C_{66} \tag{11}$$

In the same way as in radial anisotropy models, we represent radial anisotropy as

$$\xi = \frac{N}{L}. \tag{12}$$

Each aggregate is composed of 512 crystals for each of the two mineral phases. Aggregates are initially spaced by 10 km in the tangential and radial directions, respectively. Those aggregates that are found in the upper mantle are harzburgitic in composition. At first, backward advection is performed until all the upper mantle aggregates flow beneath the mantle transition zone or fall outside the lower boundary. In a second step, forward advection is computed for the same

amount of time as during backward advection, so that by the end of the D-Rex model the aggregates are homogeneously distributed as in the original configuration. LPO development is calculated during this second step when transition zone crystal aggregates transform into upper mantle aggregates at ~410 km depth.

We use the following D-Rex parameters (Kaminski and Ribe, 2001; Kaminski et al., 2004); the grain nucleation rate, λ^*=5, the grain boundary sliding parameter, χ=0.9 and a grain boundary mobility, M* of 1. Boneh et al. (2015) found that M* has the most pronounced effect on the resulting texture and that λ^* and χ have a relatively small influence that becomes negligible at low M*. When compared to parameters used in other studies (e.g., Hedjazian et al., 2017, who used M* = 10), the employed lower M* and higher χ yield weaker A-type mantle fabrics for the same amount of deformation. Fig. S4 and S5 show the results of experiments with nCRSS yielding A-type olivine fabric ([100](010) = 1; [100](001) = 2; [001](010) = 3), but with different values of M*=1, 10 and χ=0.3, 0.7, 0.9. It can be seen that by decreasing M* and increasing χ the fabric strength and anisotropy decrease. However, when azimuthal anisotropy is around 3% and comparable to observations the fabrics start to be characterized by the b and c axes forming a girdle at high angles from the shear plane, which is not consistent with observations. The only true A-type fabric is obtained with M*=10 and χ=0.3 and that is characterized by strong anisotropy. Therefore, in order to obtain AG-type-like fabrics where the a- and c-axes spin in the shear plane around the b-axis, we set the same high activities (i.e., low CRSS) for the [100](010) and [001](010) slip systems relative to that of [100](001) (Table S3).

Flow is less focused towards the ridge beneath the fast plate, as seen by the more horizontal orientations of the FSE (Fig. S12 in the supplementary materials). Moreover, at depth beneath the older region of the faster , the length of the FSE is larger, but not necessarily more horizontal than beneath the slower plate, which explains the deeper radial anisotropy.

While experimental studies suggest that pressure may influence the relative strengths of different slip systems in olivine (e.g., Li et al., 2003; Couvy et al., 2004; Raterron et al., 2004; Jung et al., 2009), the effect of pressure at Earth-like stresses and strain rates is still debated.

LPO development in the diffusion creep regime is still unclear. Although it is not expected to generate a consistent rotation of the crystallographic axes (Karato, 1993), a numerical modelling study (Wheeler, 2009) suggest LPO could be weakened but preserved in the diffusion creep regime. Specifically, (Wheeler, 2009) suggested that if microstructural changes due to diffusion creep dominate that due to grain growth (which we do not model in this study), the LPO will be weakened but preserved. Or if grain growth is rapid in comparison to deformation-induced

microstructural evolution, grain growth may indirectly aid LPO destruction although this has yet to be quantified. On the other hand, olivine deformation experiments (e.g., Miyazaki et al., 2013) have suggested that depending on the temperature and presence of melt, grain boundary sliding on new crystallography-controlled boundaries accommodated by diffusion can result in LPO. As there is no clear consensus, we follow the numerical study of Hedjazian et al. (2017) to implement fluid body rotation by calculating the bulk rotation of the LPO by multiplying the matrix of the direction cosines of the aggregates with the rotations of the FSE. In this way, we preserve and do not alter the strength of the LPO. Therefore, lithospheric anisotropy is only the result of frozen-in LPO from formerly deformed asthenospheric aggregates.

At low strain rates and stresses beneath slow plates diffusion creep will accommodate more deformation than dislocation creep and the timescale for LPO evolution will increase. However, the geodynamic models presented in this study account for this competition of creep mechanisms and still suggest that the amount of deformation accommodated by dislocation creep beneath slow moving plates is enough to create a LPO consistent with plate motion. Our estimates are likely an upper bound as the models do not consider pre-existing fabrics, they are kinematically driven and thus the imposed plate velocity creates larger stresses, and they are 2D models such that no lateral flow is possible and lateral small-scale convection currents cannot form. However, when increasing the relative contribution of diffusion to dislocation creep (e.g., Hedjazian et al., 2017) LPO still forms.

S1.3 Tomographic filtering

Following a similar approach to Ritsema et al., 2007 and Styles et al., 2011, in tomography we typically relate seismic data $\mathbf{d}$ to a model $\mathbf{m}$ of wave speed variation in Earth in a linear fashion

$$\mathbf{Gm} = \mathbf{d}, \tag{13}$$

and estimate $\mathbf{m}$ by damped least-squares inversion (e.g., Tarantola, 1987; Menke, 1989; Scales et al., 2001; Aster et al., 2005) to minimise

$$\Gamma(\mathbf{m}) = (\mathbf{Gm} - \mathbf{d})^T(\mathbf{Gm} - \mathbf{d}) + \epsilon\mathbf{m}^T\mathbf{m}, \tag{14}$$

where ϵ is a damping parameter. The solution to (5) is

$$\mathbf{m}^\dagger = \mathbf{G}^\dagger\mathbf{d}, \tag{15}$$

where $\mathbf{G}^\dagger$ is the generalized inverse of $\mathbf{G}$. If $\mathbf{U}\Lambda\mathbf{U}^T$ is the eigenvalue decomposition of $\mathbf{G}^T\mathbf{G}$, we can define the generalized inverse by

$$\mathbf{G}^\dagger = \mathbf{U}\Lambda^{-1}\mathbf{U}^T\mathbf{G}^T, \tag{16}$$

where $\Lambda^{-1} = (\Lambda + \epsilon I)^{-1}$. Combining (5) and (7) yields

$$\mathbf{m}^{\dagger} = \Re \mathbf{m}^{t}, \tag{17}$$

where we have defined the resolution filter $\Re = \mathbf{G}^{\dagger}\mathbf{G}$ that specifies how the geoydnamical model $\mathbf{m}^{t}$ is mapped into the filtered geodynamical model, $\mathbf{m}^{\dagger}$. The spatially heterogeneous resolution, an attribute of any tomographic model, is fully described by $\Re$.

The resolution filter for SGLOBE-rani is built from the large data set used to construct SGLOBE-rani, which comprises Rayleigh- and Love-wave phase velocity, group velocity and teleseismic body-wave traveltime measurements (see Chang et al., 2015 for more details). It reflects the regularization choices as well as forward and inverse modelling approach used to build SGLOBE-rani. Hence, applying the resolution filter to the input geodynamical model $\mathbf{m}^{t}$ corresponds to performing a synthetic inversion test with $\mathbf{m}^{t}$ as the input model, and performing the inversion in exactly the same conditions as those used to build SGLOBE-rani. We have tested the effect of different levels of regularization on the flattening of a depth-age trend, however, future studies should investigate other factors such as data coverage and resolution.

References

Aster, R. C., Borchers, B., and Thurber, C. H. (2005). *Parameter Estimation and Inverse Problems.* Elsevier, New York.

Auer, L., Boschi, L., Becker, T., Nissen-Meyer, T., and Giardini, D. (2014). Savani: A variable resultion whole-mantle model of anisotropic shear velocity variations based on multiple data sets. *Journal of Geophysical Research: Solid Earth*, 119:3006–3034.

Boneh, Y., Morales, L. F., Kaminski, É., and Skemer, P. (2015). Modeling olivine CPO evolution with complex deformation histories: Implications for the interpretation of seismic anisotropy in the mantle. *Geochemistry Geophysics Geosystems*, 16:3436–3455.

Browaeys, J. T. and Chevrot, S. (2004). Decomposition of the elastic tensor and geophysical applications. *Geophysical Journal International*, 159:667–678.

Chang, S.-J., Ferreira, A. M. G., Ritsema, J., van Heijst, H. J., and Woodhouse, J. H. (2015). Joint inversion for global isotropic and radially anisotropic mantle structure including crustal thickness perturbations. *Journal of Geophysical Research: Solid Earth*, 120(6):4278–4300.

Couvy, H., Frost, D. J., Heidelbach, F., Nyilas, K., Ungár, T., Mackwell, S., and Cordier, P.

(2004). Shear deformation experiments of forsterite at 11 GPa - 1400C in the multianvil apparatus. *European Journal of Mineralogy*, 16(6):877–889.

DeMets, C., Gordon, R. G., and Argus, D. F. (2010). Geologically current plate motions. *Geophysical Journal International*, 181(1):1–80.

Evans, B. and Goetze, C. (1979). The temperature variation of hardness of olivine and its implication for polycrystalline yield stress. *Journal Geophysical Research*, 84:5505–5524.

Faccenda, M. and Capitanio, F. A. (2013). Seismic anisotropy around subduction zones: Insights from three-dimensional modeling of upper mantle deformation and SKS splitting calculations. *Geochemistry, Geophysics, Geosystems*, 14(1):243–262.

Gerya, T. V., Connolly, J. A., and Yuen, D. A. (2008). Why is terrestrial subduction one-sided? *Geology*, 36(1):43–46.

Gerya, T. V. and Yuen, D. A. (2003). Characteristics-based marker-in-cell method with conservative finite-differences schemes for modeling geological flows with strongly variable transport properties. *Physics of the Earth and Planetary Interiors*, 140:293–318.

Hedjazian, N., Garel, F., Davies, D. R., and Kaminski, É. (2017). Age-independent seismic anisotropy under oceanic plates explained by strain history in the asthenosphere. *Earth and Planetary Science Letters*, 460:135–142.

Hirth, G. and Kohlstedt, D. L. (2003). Rheology of the Upper Mantle and the Mantle Wedge: A View from the Experimentalists. *Geophysical Monograph Series*, 138:83–105.

Jung, H., Mo, W., and Green, H. W. (2009). Upper mantle seismic anisotropy resulting from pressure-induced slip transition in olivine. *Nature Geoscience*, 2(1):73–77.

Kaminski, É. and Ribe, N. M. (2001). A kinematic model for recrystallization and texture development in olivine polycrystals. *Earth and Planetary Science Letters*, 189:253–267.

Kaminski, É., Ribe, N. M., and Browaeys, J. T. (2004). D-Rex, a program for calculation of seismic anisotropy due to crystal lattice prefferred orientation in the convective upper mantle. *Geophysical Journal International*, 158:744–752.

Karato, S.-I. (1993). Importance of anelasticity in the interpretation of seismic tomography. *Geophysical Research Letters*, 20(15):1623–1626.

Karato, S.-I. and Wu, P. (1993). Rheology of the upper mantle: A synthesis. *Science*, 260(5109):771–778.

Katayama, I. and Karato, S.-I. (2008). Low-temperature, high-stress deformation of olivine under water-saturated conditions. *Physics of the Earth and Planetary Interiors*, 168:125–133.

Kustowski, B., Ekström, G., and Dziewoński, A. M. (2008). Anisotropic shear-wave velocity structure of the Earth's mantle: A global model. *Journal of Geophysical Research: Solid Earth*, 113:B06306.

Li, L., Raterron, P., Weidner, D., and Chen, J. (2003). Olivine flow mechanisms at 8 gpa. *Physics of the Earth and Planetary Interiors*, 138(2):113–129.

Menke, W. (1989). *Geophysical Data Analysis: Discrete Inverse Theory.* Elsevier, New York.

Mishin, Y. A., Gerya, T. V., Burg, J.-P., and Connolly, J. A. (2008). Dynamics of double subduction: Numerical modeling. *Physics of the Earth and Planetary Interiors*, 171(1):280–295. Recent Advances in Computational Geodynamics: Theory, Numerics and Applications.

Miyazaki, T., Sueyoshi, K., and Hiraga, T. (2013). Olivine crystals align during diffusion creep of Earth's upper mantle. *Nature*, 502:321–325.

Montagner, J.-P. and Nataf, H.-C. (1986). A simple method for inverting the azimuthal anisotropy of surface waves. *Journal of Geophysical Research*, 91(B1):511–520.

Rappisi, F. and Faccenda, M. (2019). Geodynamic and seismological numerical modelling for seismic anisotropy studies. American Geophysical Union Fall Meeting.

Raterron, P., Wu, Y., Weidner, D. J., and Chen, J. (2004). Low-temperature olivine rheology at high pressure. *Physics of the Earth and Planetary Interiors*, 145(1):149–159.

Ritsema, J., McNamara, A. K., and Bull, A. L. (2007). Tomographic filtering of geodynamic models: Implications for models interpretation and large-scale mantle structure. *Journal of Geophysical Research: Solid Earth*, 112:1–8.

Scales, J. A., Smiths, M. L., and Treitel, S. (2001). *Introductory Geophysical Inverse Theory.* Samizdat.

Sleep, N. H. (1990). Hotspots and Mantle Plumes: Some Phenomenology. *Journal of Geophysical Research*, 95(B5):6715–6736.

Styles, E., Goes, S., Keken, P. E. V., Ritsema, J., and Smith, H. (2011). Synthetic images of dynamically predicted plumes and comparison with a global tomographic model. *Earth and Planetary Science Letters*, 311:351–363.

Tarantola, A. (1987). *Inverse Problem Theory.* Elsevier, New York.

Wheeler, J. (2009). The preservation of seismic anisotropy in the Earth's mantle during diffusion creep. *Geophys. J. Int.*, 178:1723–1732.

Table S1: Reference physical parameters used in I2VIS 2D ridge flow simulations. *Evans and Goetze (1979). R=8.313 J mol^{-1}K^{-1} is the gas constant, μ=80 GPa is the shear modulus, b=0.5 nm is the Burgers vector, d=1 mm is the grain size.

**Quantity**	**Symbol**	**Units**	**Value**
**Dislocation Creep (Karato and Wu, 1993)**			
Prefactor	A	s^{-1}	3.5x10^{22}
Activation energy	E	kJ mol^{-1}	540
Activation volume	V	cm^{3}mol^{-1}	20
Stress exponent	n	-	3.5
Grain-size exponent	m	-	0
**Diffusion Creep (Karato and Wu, 1993)**			
Prefactor	A	s^{-1}	8.7x10^{15}
Activation energy	E	kJ mol^{-1}	300
Activation volume	V	cm^{3}mol^{-1}	6
Stress exponent	n	-	1
Grain-size exponent	m	-	2.5
**Peierls Creep (Katayama and Karato, 2008)**			
Prefactor	A	Pa^{2}s	10$^{7.8}$
Activation energy	E	kJ mol^{-1}	532
Activation volume	V	cm^{3}mol^{-1}	12
Peierls stress*	$\sigma_{Peierls}$	GPa	9.1
Exponent	p,q	-,-	1,2

Table S2: Summary of the rheological properties for all rock types: rock type, depth extent, minimum viscosity, maximum viscosity, prefactor, power-law exponent, activation energy, activation volume, coefficient of friction at zero deformation and at strain ϵ_1 (from left to right) used in the 2D ridge-to-slab model. Note that the rheological parameters used to compute viscous behaviour in mantle rocks are defined in Table S1.

**Rock type**	Depth extent (km)	η_{min} (Pa s)	η_{max} (Pa s)	A_{D} (Pa s)	n	E (J)	V (J bar^{-1})	μ_0	μ_1	ε_1
Sticky Air	0-25	1×10^{18}	1×10^{18}	-	-	-	-	-	-	-
Water	25-30	1×10^{18}	1×10^{18}	-	-	-	-	-	-	-
Oceanic crust basalt	30-32	1×10^{18}	1×10^{25}	4.8×10^{22}	3.2	2.38×10^{5}	0.8	0.05	0.05	0.5
Oceanic crust gabbros	32-37	1×10^{18}	1×10^{25}	4.8×10^{22}	3.2	2.38×10^{5}	1.0	0.15	0.15	0.0
Lithosphere	37-120	1×10^{18}	1×10^{25}	-	-	-	-	0.6	0.4	0.5
Shear Zone	30-120 (20km thick)	1×10^{18}	1×10^{25}	5.01×10^{20}	4	4.7×10^{5}	1.2	0.05	0.05	0.5

Table S3: Resolved shear stresses, grain boundary mobility (M*), grain boundary sliding parameter (χ) and grain nucleation rate (λ*) of the olivine slip systems for both AG-type (Rappisi and Faccenda, 2019) and A-type (Kaminski et al., 2004) fabric. Creep parameters are defined in Table S1.

**Olivine Fabric**	(010)[100]	(001)[100]	(010)[001]	(100)[001]	M*	χ	λ*
AG-type	1	5	1	∞	1	0.9	5
A-type	1	2	3	∞	10	0.3	5

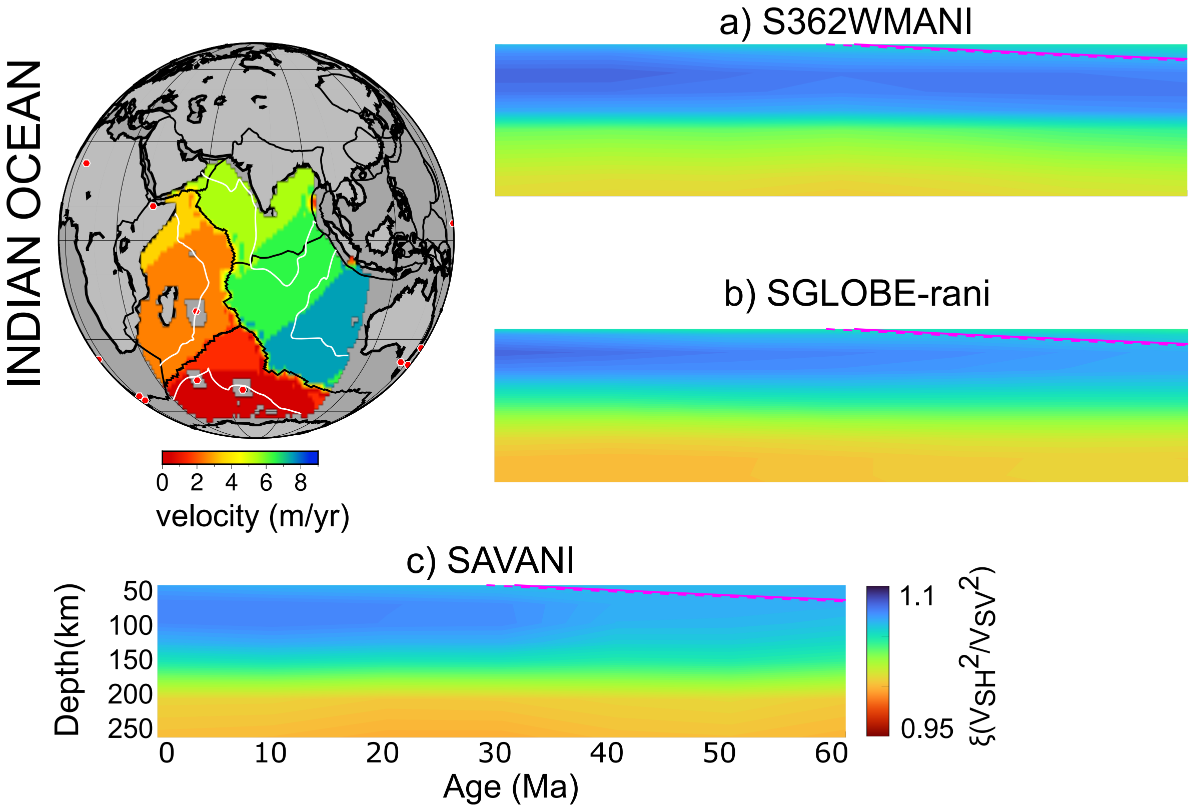

Figure S1: Radial anisotropy in a) S362WMANI (Kustowski et al., 2008), b) SGLOBE-rani (Chang et al., 2015) and c) SAVANI (Auer et al., 2014) as a function of ocean-sea floor age beneath the Indian ocean for profiles within the white lines (up to 60 Ma) in the map of observed plate motion (NUVEL-1A in a no-net-rotation frame from DeMets et al., 2010). Profiles are removed 3 degrees each side of each plume (red circles) in the Sleep (1990) hotspot list. The 1000°C isotherm from the half-space cooling model is shown by a dashed/solid magenta line.

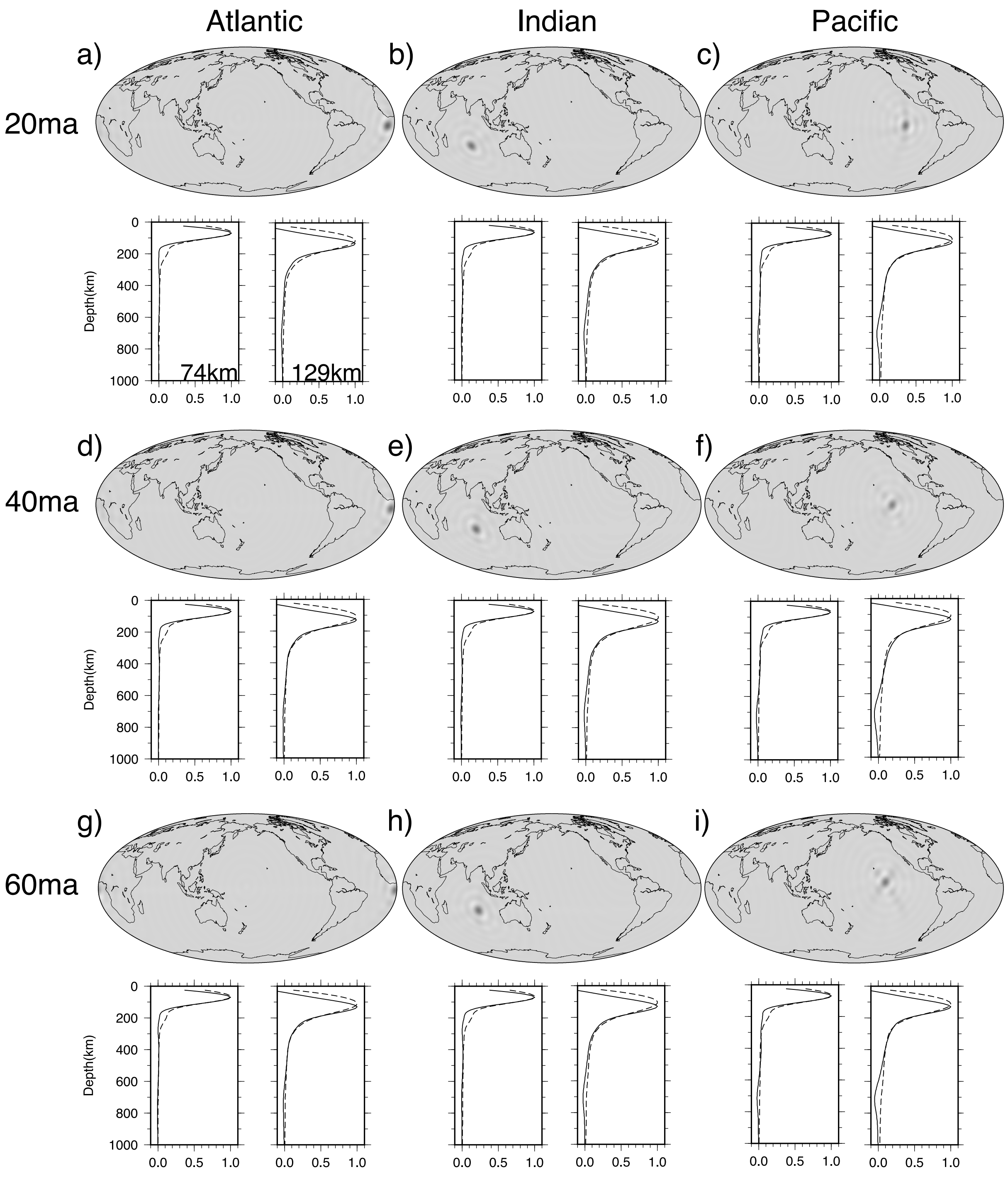

Figure S2: Backus-Gilbert resolution kernels for SGLOBE-rani. The first column of each set of kernels shows map views of the anisotropic kernels at 74 km depth. The second row presents the radial dependence of the kernels. Solid and dashed lines represent kernels for isotropy and radial anisotropy, respectively. The kernels are calculated beneath 20Ma (a-c), 40Ma (d-f) and 60Ma (g-i) Atlantic, Indian and Pacific lithosphere at 74 and 129 km depth. These kernels describe how the velocity or anisotropy perturbation at a given point is the spatial average of real structure. As expected, isotropic structure is better resolved than anisotropy, but the latter is still relatively well resolved.

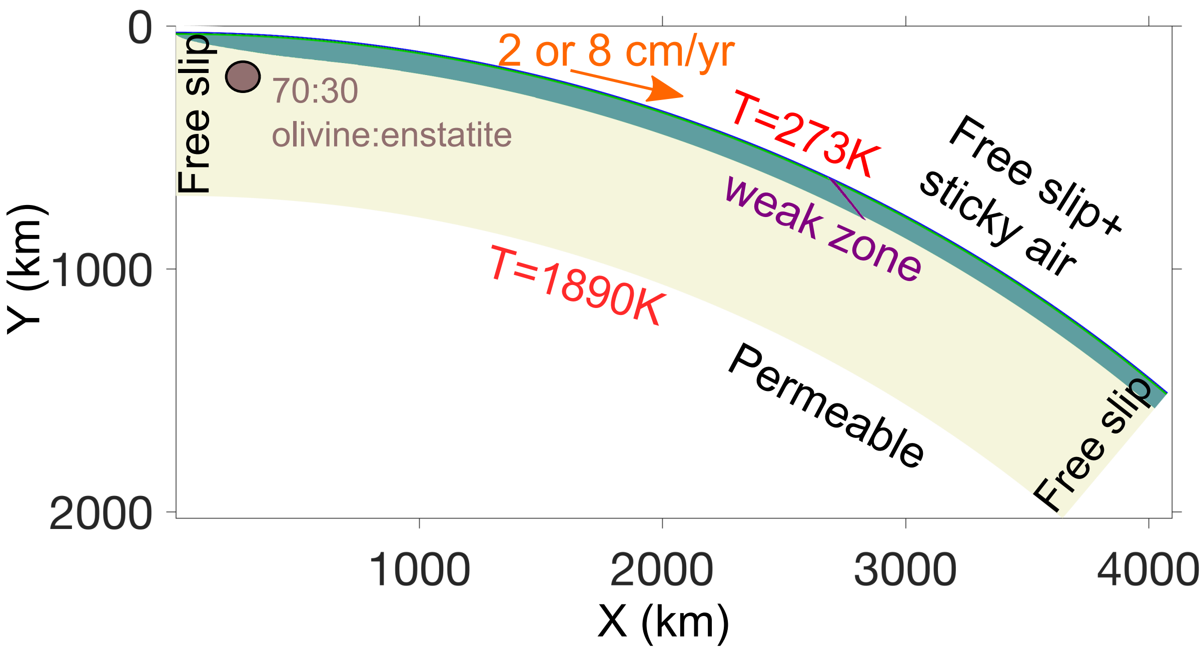

Figure S3: Initial 2D ridge flow simulation domain. Mechanical (black and orange text) and thermal (red text) boundary conditions are indicated. Left and right boundaries are free slip with the bottom boundary being permeable. We push the plate with 2 or 8/,cm/yr. For the model with a higher plate velocity we extend the model from 40 to 80° and move the weak zone accordingly. The model consists of a 25 km sticky air (white), 5 km water (blue), 2 km basalt (dark green), 5 km gabbros (light green), lithosphere (turquoise), asthenosphere (beige). A 20 km thick weak zone (purple) is also included to aid subduction of oceanic lithosphere. We calculate seismic anisotropy from the ridge to beneath 60 Ma lithosphere which is not affected by subduction-induced flow and down to 400 km with a 70:30 olivine, enstatite assemblage.

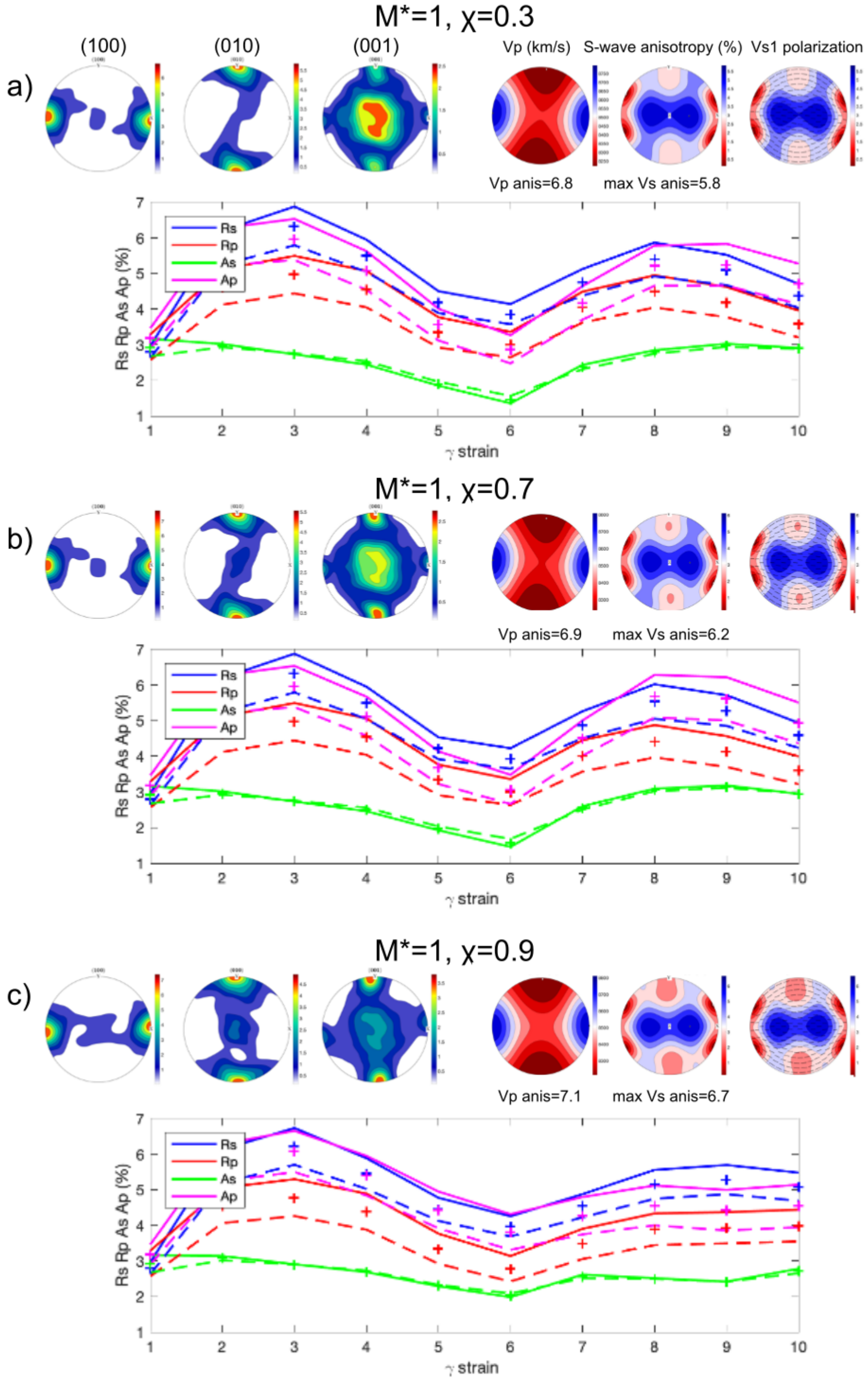

Figure S4: Experiments with nCRSS yielding A-type olivine fabric ([100](010) = 1; [100](001) = 2; [001](010) = 3), grain boundary mobility M*=1 and grain boundary sliding χ= a) 0.3, b) 0.7 and c) 0.9. Top: Pole projections of the Olivine CPO, Vp and dVs (after Voigt avgeraging) as a function of the wave propagation and plotted with MTEX, at shear strain γ=10. Bottom: Evolution of radial and azimuthal P and S-wave anisotropies as a function of strain and calculated for different averaging schemes of the single crystal elastic properties (continuous lines: Voigt avg; dashed lines: Reuss avg; crosses: Hill avg). By increasing χ the fabric strength and anisotropy decrease.

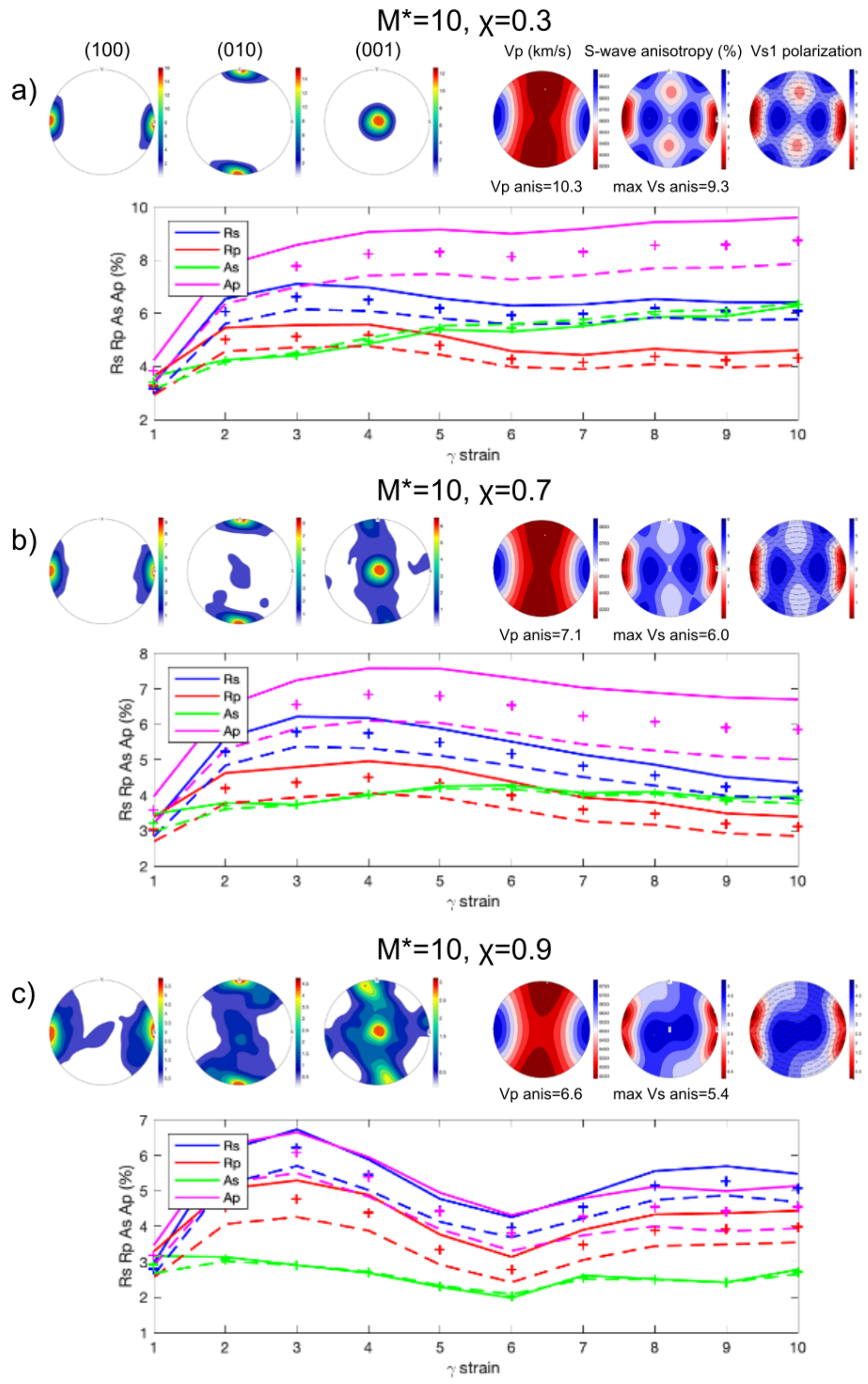

Figure S5: Experiments with nCRSS yielding A-type olivine fabric ([100](010) = 1; [100](001) = 2; [001](010) = 3), grain boundary mobility M*=10 and grain boundary sliding χ= a) 0.3, b) 0.7 and c) 0.9. Top: Pole projections of the Olivine CPO, Vp and dVs (after Voigt avgeraging) as a function of the wave propagation and plotted with MTEX, at shear strain γ=10. Bottom: Evolution of radial and azimuthal P and S-wave anisotropies as a function of strain and calculated for different averaging schemes of the single crystal elastic properties (continuous lines: Voigt avg; dashed lines: Reuss avg; crosses: Hill avg). By increasing M*=10 the fabric strength and anisotropy decrease.

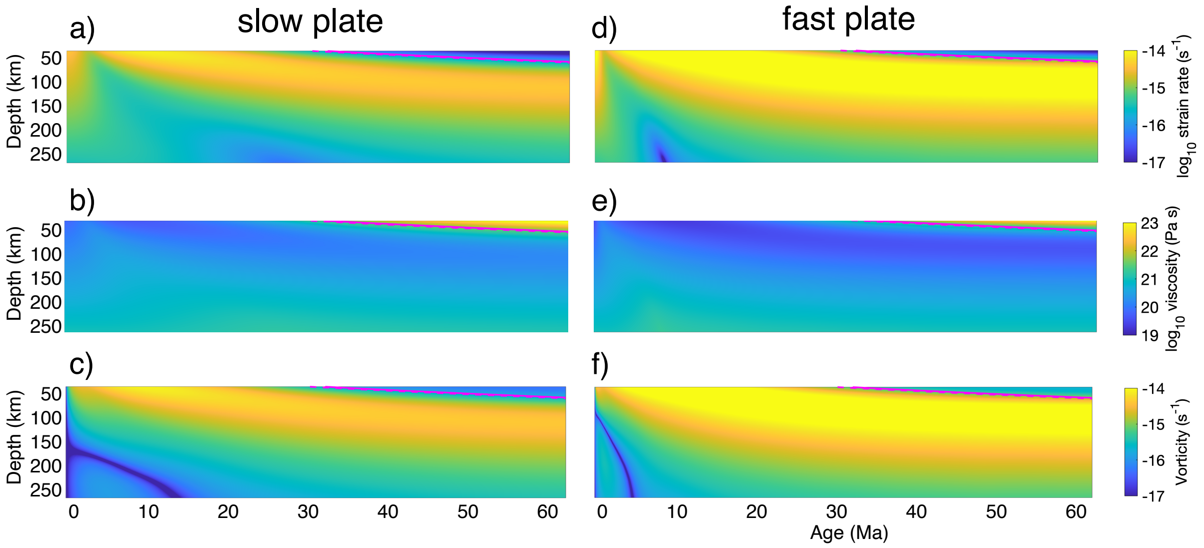

Figure S6: a) Strain rate, b) viscosity and c) vorticity magnitude for the slow plate model (plate velocity 2 cm/yr). d-f) Same as a-c) but for the fast plate (plate velocity 8 cm/yr) with the rheological parameters found in Table S1. By comparing strain rate ($\dot{\varepsilon}_{II}$) and vorticity (Ω_{II}) maps it is evident that plate spreading is accommodated by mostly pure shear deformation (vorticity number, $\Gamma = \Omega_{II}/\dot{\varepsilon}_{II} \sim 0$), while away from the ridge sub-horizontal simple shear deformation dominates ($\Gamma \sim 1$). The 1000°C isotherm from the half-space cooling model is shown by the dashed/solid magenta line.

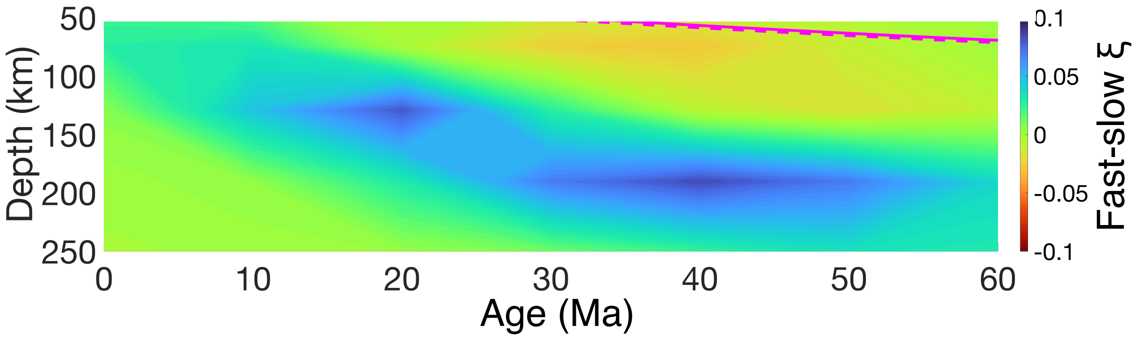

Figure S7: The difference in the predicted radial anisotropy beneath fast and slow model presented in this study, before tomographic filtering.

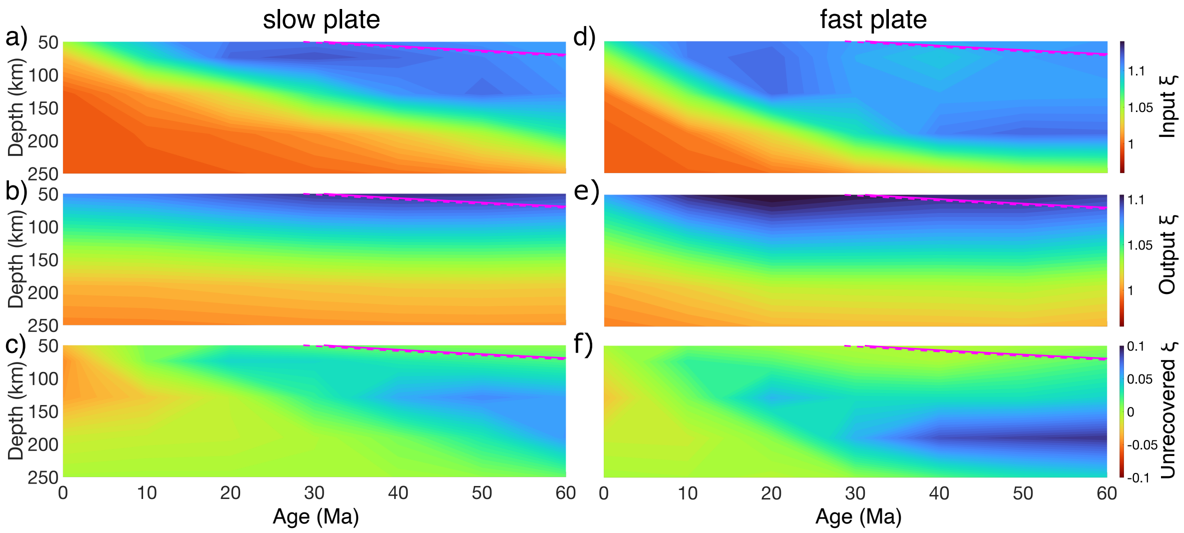

Figure S8: Predictions of radial anisotropy as a function of ocean-sea floor age from the 2D-ridge flow model with a slow plate (2 cm/yr) which is as used an an input for the tomographic filtering. b) Tomographically filtered model with a) as the input and using the same source-receiver configuration and procedure as that used to build SGLOBE-rani. c) The unrecovered radial anisotropy. d-f) Same as a-c) but for the fast plate (8 cm/yr). The 1000°C isotherm from the half-space cooling model is shown by the dashed/solid magenta line.

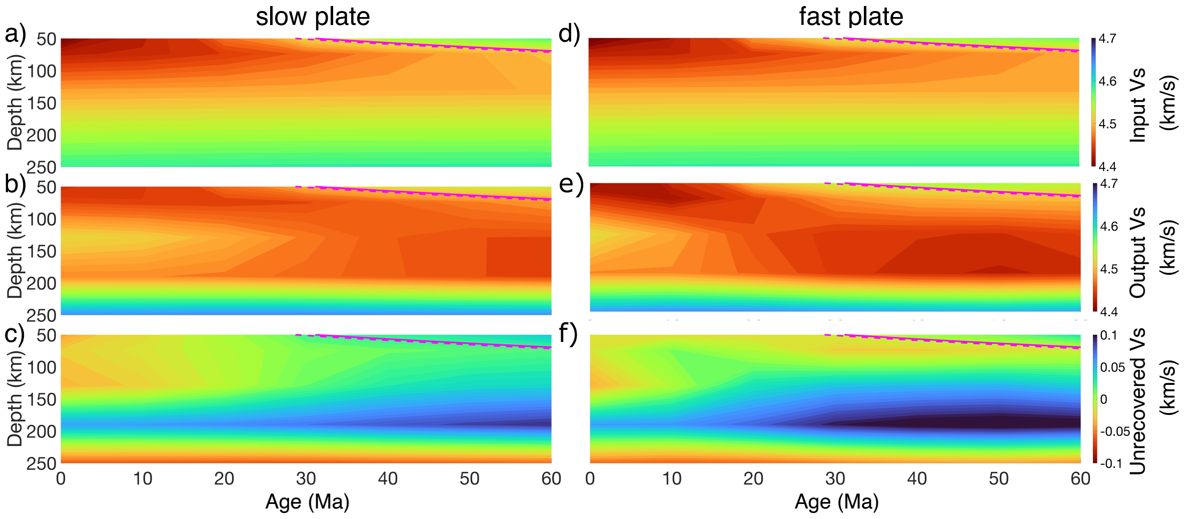

Figure S9: a) Predictions of isotropic shear wave velocity as a function of ocean-sea floor age from the 2D-ridge flow model with a slow plate (2 cm/yr) which is as used an an input for the tomographic filtering. b) Tomographically filtered model with a) as the input and using the same source-receiver configuration and procedure as that used to build SGLOBE-rani. c) The unrecovered isotropic shear wave velocity. d-f) Same as a-c) but for the fast plate (8 cm/yr). The 1000°C isotherm from the half-space cooling model is shown by the dashed/solid magenta line.

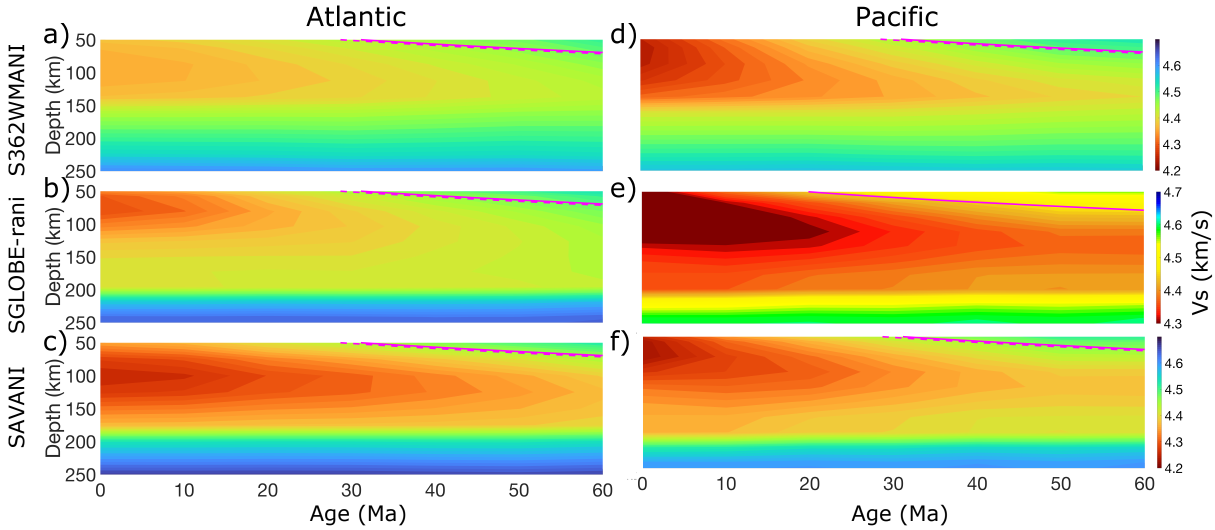

Figure S10: a) Isotropic shear wave velocity as a function of ocean-sea floor age beneath the Atlantic for a) S362WMANI, b) SGLOBE-rani and c) SAVANI. d-f) Same as a-c) but for the Pacific. The 1000°C isotherm from the half-space cooling model is shown by the dashed/solid magenta line.

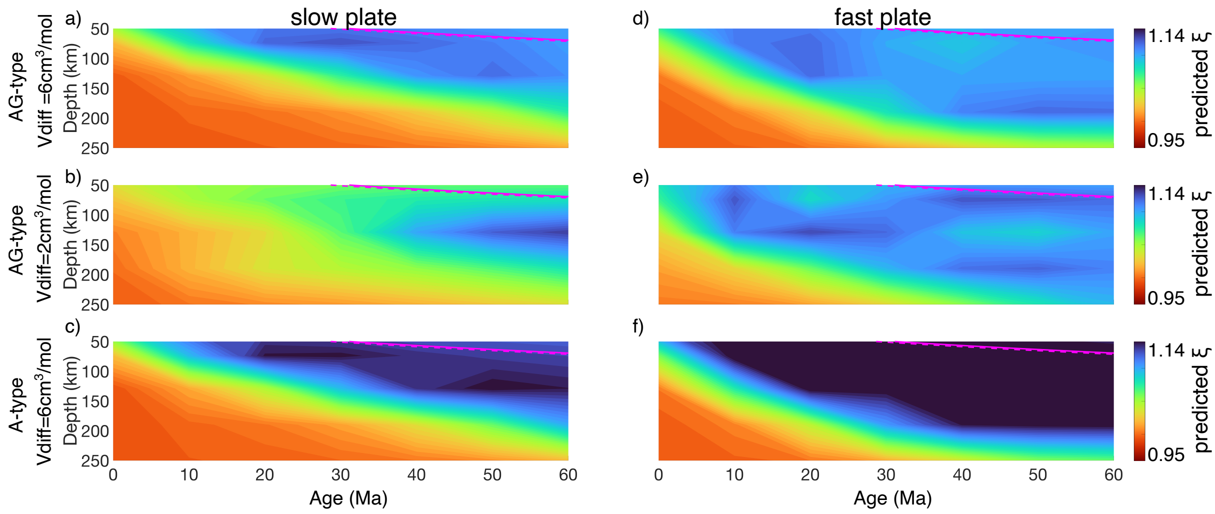

Figure S11: a) Predictions of radial anisotropy as a function of ocean-sea floor age from the 2D-ridge flow model for the slow plate (2 cm/yr) for with the rheological parameters found in Table S1 (with $V_{\text{diffusion}} = 6\,\text{cm}^3/\text{mol}$ and AG-type fabrics). b) Same as a) but with $V_{\text{diffusion}} = 2\,\text{cm}^3/\text{mol}$. c) Same as a) but with A-type fabrics. d-f) Same as a-c) but for the fast plate (8 cm/yr). The 1000°C isotherm from the half-space cooling model is shown by the dashed/solid magenta line.

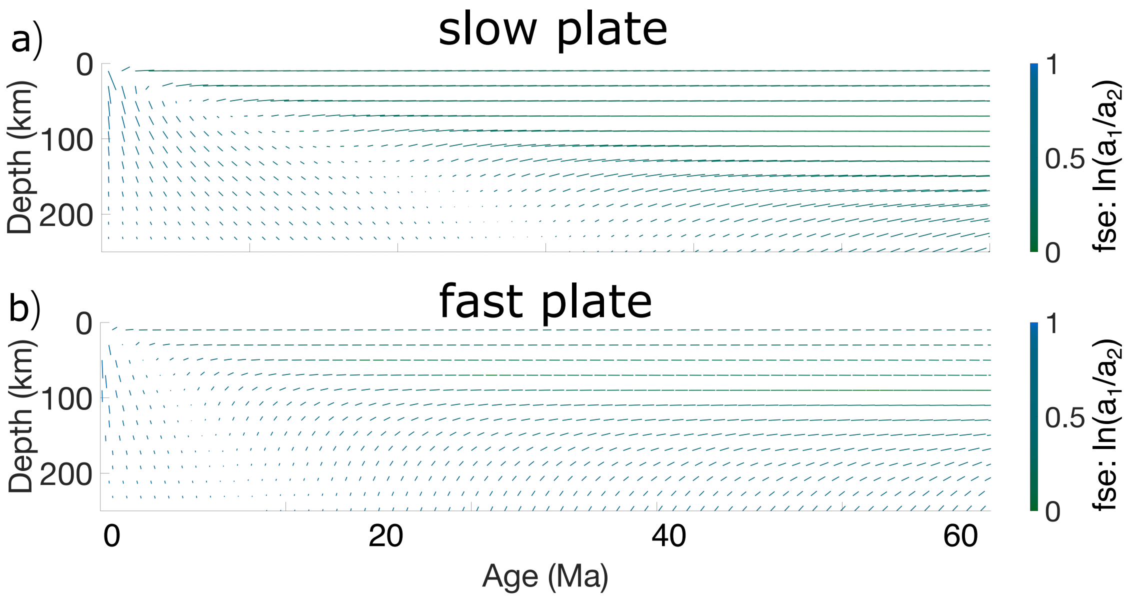

Figure S12: 2-D representation of the finite strain ellipsoid (FSE) beneath the a) slow and b) fast plate for the reference model with the rheological parameters found in Table S1. The orientations of the maximum (a_1) FSE axis for the mantle aggregates where the bar length is proportional to $\ln(a_1/a_2)$ as well as the color scale.
